# Flow cytometry for screening and prioritisation of urine samples: a retrospective comparison with culture

**DOI:** 10.1186/s12879-025-11374-8

**Published:** 2025-07-30

**Authors:** Vicky Sender, John Kerr White, Ludvig Bolinder, Karin Amilon, Mirja Hägg, Karin Haij Bhattarai, Niklas K. Björkström, Baharak Saeedi

**Affiliations:** 1 Department of Clinical Microbiology, Karolinska University Hospital, Stockholm, Sweden; 2 Center for Infectious Medicine, Department of Medicine Huddinge, Karolinska Institutet, Karolinska University Hospital, Stockholm, Sweden; 3 Department of Microbiology, Tumor and Cell Biology, Karolinska Institutet, Stockholm, Sweden; 4 Department of Infectious Diseases, Karolinska University Hospital, Stockholm, Sweden; 5 Division of Clinical Microbiology, Department of Laboratory Medicine, Karolinska Institutet, Stockholm, Sweden

**Keywords:** Bacteriuria, Flow cytometry, Rapid diagnostics, Screening, Urine culture, Urinary tract infection

## Abstract

**Background:**

Efficient urinary tract infection (UTI) diagnostics require reliable methods to rapidly exclude negative samples while accurately identifying positive cases. Automated urine flow cytometry (UFC) offers a promising approach to streamline laboratory workflows by reducing unnecessary cultures and providing early bacterial classification. This study evaluated the performance of a UFC-based screening method compared to conventional urine culture.

**Methods:**

4005 urine samples were analysed using UFC (Sysmex UF-5000) and compared to culture results. Receiver operating characteristic (ROC) curve analyses assessed method agreement across different patient subpopulations. Predictive values were calculated for the total population and the ten different subpopulations at different cut-offs. Carryover and cross-contamination were also examined.

**Results:**

Based on our data, we propose an algorithm using bacterial count from UFC to guide urine culture decisions. A cut-off of < 30 BACT cells/µl to support exclusion of negative samples (AUC 0.921) could potentially reduce culture rates by ~ 32%. Due to reduced diagnostic performance in subgroup analyses, pregnant women and children were excluded from the rule-out strategy. UFC also demonstrated high sensitivity and specificity in identifying samples likely to be culture-positive, and a cut-off of > 4000 BACT cells/µl was suggested to indicate clinically relevant bacteriuria (AUC 0.917). Pregnant women were excluded from this rule-in approach due to limited discriminatory performance in this subgroup. Additionally, the BACT-Info flag “Gram Neg?” predicted Gram-negative bacteria in 61% of culture-positive samples, with 96% concordance, offering potential early indication of Gram-negative infections.

**Conclusions:**

UFC shows potential as a useful screening tool for reducing unnecessary cultures by helping to exclude negative samples and highlighting those likely to be positive. The “Gram Neg?” flag facilitated early differentiation of Gram-negative infections, aiding timely targeted antibiotic therapy. Thus, implementing UFC-based screening in routine diagnostics could reduce laboratory workload while maintaining diagnostic accuracy and patient safety.

**Supplementary Information:**

The online version contains supplementary material available at 10.1186/s12879-025-11374-8.

## Background

Urinary tract infections (UTIs) are among the most common bacterial infections worldwide with about 400 million cases yearly and over 250,000 associated deaths making it one of the most common bacterial diseases and drivers of antimicrobial usage [1]. Accurate and timely diagnosis is crucial for guiding appropriate treatment and antimicrobial stewardship.

Urine culture remains a cornerstone in diagnosing UTIs, providing microbiological confirmation of the causative bacteria. However, this method is labour-intensive and time-consuming, requiring 24 to 48 h for final reporting. Furthermore, a significant proportion of cultured urine samples yield no or insignificant bacterial growth [2]. Empirical treatment of suspected UTIs often begins before confirmation by culture, potentially leading to unnecessary antibiotic use and increased bacterial resistance. This highlights the need for an efficient screening method to optimise sample processing and reduce unnecessary laboratory workload.

Over the past two decades, fully automated urine analysis instruments that detect parameters such as bacteria, leukocytes, yeast, erythrocytes, and epithelial cells, have been developed to improve the efficiency of urine diagnostics. While traditional urinalysis offers a high negative predictive value, its diagnostic utility is limited by a low positive predictive value, subjective interpretation, and inability to differentiate or quantify bacterial presence accurately. Novel screening technologies, such as flow cytometry, are valuable additions for improving bacteriuria detection and reducing diagnostic turnaround time [3]. Numerous European laboratories have implemented urine flow cytometry (UFC) as part of their urine routine diagnostics [4–10]. UFC enables rapid screening for the identification of urine samples with no or insignificant bacterial concentrations, allowing reporting of negative results up to 48 h earlier than conventional culture. This faster turnaround benefits both patients and the healthcare system by reducing excess costs, unnecessary antibiotic use, and prolonged hospital stays [11]. UFC is recognised as a rapid, accurate, and reliable screening method that significantly reduces excessive urine cultures in microbiology laboratories [4, 6–9, 12, 13].

Beyond exclusion, there is growing interest in using UFC to predict positive urine samples, allowing for prioritised processing and rapid diagnostic reports from the lab to the clinic. This predictive capability helps laboratories streamline workflow, reduce manual steps, and speed up clinical decisions [4, 5, 9, 10, 13, 14].

This study aimed to evaluate the Sysmex UF-5000 as a screening tool for optimising urine sample processing in the laboratory. Specifically, we assessed its ability to differentiate between negative and likely positive samples, thereby reducing unnecessary cultures, minimising manual workload, and improving turnaround time. Subgroup analyses were performed to evaluate the method’s reliability across different patient populations. Carryover and cross-contamination studies were also performed to assess optimum rinse modes during UFC analysis.

## Methods

### Urine sample and patient data collection

A total of 4295 urine samples were received at the Department of Clinical Microbiology, Karolinska University Hospital in Stockholm, Sweden, from both hospitalised patients and outpatients during the study period. Of these, 290 samples collected via catheter or nephrostomy were excluded to ensure consistency in sampling method and to reduce potential variability in pre-analytical conditions. The final analysis included 4005 midstream urine samples collected according to local standard procedures across a range of clinical settings, from primary care centres to intensive care units. Midstream collection is the recommended approach to minimise contamination risk, although some variation in sample handling may occur between settings. Samples were collected in non-preservative tubes, transported, and stored at 4–8 °C before analysis. Of the total 4295 samples, 4005 were from midstream urine, 281 from catheters, and 9 from nephrostomies. We collected patient data regarding age (in years), sex, hospitalised or outpatient status, and pregnancy status (Table 1).

**Table 1:** Characteristics of the study population (n = 4295)

Characteristics of the study population	Number, n (%)
Age, mean (range), years	56 (0–103)
*Sex*	
Female	2773 (65)
Male	1522 (35)
Children (≤ 15 years)	313 (7)
*Patient category*	
Hospitalised patients	1798 (42)
Outpatients	2497 (58)
Pregnant women	434 (10)
*Sample collection method*	
Midstream urine	4005 (93)
Catheter	281 (7)
Nephrostomy	9 (0.2)

### Routine microbiological analyses

All urine samples were cultivated according to our standard procedure. 10 µl of urine was inoculated on Blood agar/Brilliance UTI (BUTI) clarity agar (Oxoid) plates by an automated microbiology plater instrument InoqulA (BD Kiestra). The agar plates were incubated at 35 °C for 16 h prior to subsequent analyses. Representative colonies of dominant bacteria were identified on a matrix-assisted laser desorption/ionisation time-of-flight mass spectrometer (MALDI-TOF MS; Biotyper Sirius One, Bruker). Samples were analysed by experienced and trained technicians and interpreted according to Swedish guidelines [15]. Relevant bacteriuria was defined as growth of an uropathogen at concentrations ≥ 10^4^ CFU/mL (10^4^–10^5^ CFU/mL and > 10^5^ CFU/mL). Urine samples with no growth and no significant growth (bacterial growth < 10^4^ CFU/mL) were categorised as negative urine samples (no growth, growth of urethral flora, or small amounts of mixed culture).

Samples with uropathogenic growth at concentrations < 10^4^ CFU/mL (10^3^–10^4^ CFU/mL and 10^2^–10^3^ CFU/mL) can be relevant to report and depending on given clinical information (UTI symptoms, recurrence) antimicrobial susceptibility testing might be required. Samples with uropathogenic growth at concentrations < 10^4^ CFU/mL (10^2^–10^3^ CFU/mL and 10^3^–10^4^ CFU/mL) were reported as positive in accordance with routine practice and antimicrobial susceptibility testing (AST) was performed when clinical information indicated relevance, such as current UTI symptoms and recurrent infections. Urine samples withor more species were categorised as mixed culture [15, 16].

### Flow cytometry analysis

Urine samples were analysed by flow cytometry using the UF-5000 from Sysmex. This single diagnostic method, based on nucleic acid fluorescence staining and semiconductor laser flow cytometry, provides assessment of multiple parameters, including amount of red and white blood cells as well as number of bacteria and BACT-Info. BACT-Info categorises results as ‘Gram-positive?’, ‘Gram-negative?’, ‘Gram-pos/neg?’ (GP/GN), or ‘Unclassified’. For our analysis, we primarily used the bacterial count (BACT cells/µl) and the BACT-Info classification, and in some cases also considered the white blood cell count. The UF-5000 has a theoretical maximum throughput of 105 urine samples per hour [17]. The throughput decreases with the number of automated rinsing cycles between samples. Here, we used rinse mode 2-4-4, evaluated as optimal to avoid carryover and cross-contamination (see below).

### Carryover analysis

The automated rinsing between samples can be programmed to reduce the risk of sample-to-sample carryover. We validated six rinse programs with varying number of rinses between samples depending on the number of bacteria (Table S1). Sample-to-sample carryover was analysed by triplicate measurement in a total of 25 samples with high amounts of bacteria (> 10^5^ CFU/ml) followed by triplicate measurement of sterile saline. The carryover rate was calculated by the formula: Carryover = (blank1-blank3/sample3-blank3) × 100% for all runs and mean values were calculated. The UF-5000 instrument uses a reusable probe to aspirate urine from sample vials. According to the manufacturer, cross-contamination, i.e. transfer of cells or particles from one sample tube to the next, might occur, although the probe is washed between succeeding samples [17]. The level of probe washing is constant and independent of the rinsing of the tube system in the instrument, as described above regarding sample-to-sample carryover. Cross-contamination, defined as transfer of bacteria from one sample to the next, was assessed by measuring a urine sample with high bacterial content (> 10^5^ CFU/ml) followed by 3 samples of sterile saline. All samples were then cultured to detect if there is cross-contamination. In total 25 samples with > 10^5^ CFU/ml were analysed for sample-to-sample carryover.

### Statistical analyses

The results of the Sysmex UF-5000 BACT were compared with the results from urine cultures using Receiver Operating Characteristic (ROC) curve analysis. The diagnostic performance of flow cytometry data (BACT cells/µl) was defined by the area under the ROC curve (AUC). Subgroup analyses were performed for ten different subpopulations, and AUC values were calculated for each subgroup. AUC values used to evaluate the agreement between the two methods were as follows: AUC < 0.700 = insufficient agreement, 0.700–0.800 = good agreement, AUC 0.800–0.900 very good agreement, and AUC 0.900–1.0 = excellent agreement [18, 19]. Predictive values such as sensitivity (SE), specificity (SP), positive (PPV), and negative predictive value (NPV) were calculated for the total population and for the ten different subpopulations at different cut-offs using R (version 4.0.5). The R CRAN package pROC and epiR were used for performing statistical analysis [20, 21].

## Results

### Analysis of carryover and cross-contamination

To evaluate the most appropriate automatic rinse setting of the UF-5000 for urine analysis in our laboratory, we tested in total 25 urine samples with high amounts of Gram-negative bacteria (> 10^5^ CFU/ml, culture) and 6 different rinse programs. For tested rinse modes the carryover rate varied between 0.000 and 0.043% (Table S2). This is below the reported manufacturer carryover of up to 0.05% at 1000 bacteria/μL [17]. At rinse program 2-4-4 there was a single sample with an initial bacterial amount of BACT 10^8^ cells/µl that had a carryover rate of 0.001% (BACT 1.1 × 10^3^ cells/ml). Of note the bacterial amount detected lies under the bacterial cut-off for negative samples at 3 × 10^4^ bacteria/ml. In 2/75 sterile samples cultured, one bacterial colony grew on the agar plate (Table S3). This minimal growth is considered negligible and does not indicate clinically relevant carryover or contamination.

### Diagnostic performance of UFC compared to urine culture to support exclusion of negative samples

**Fig. 1:**
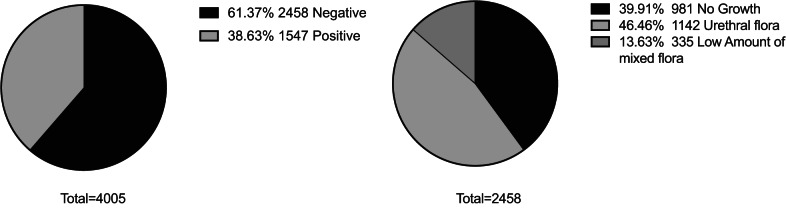
Distribution of urine culture results of the total population (n = 4005). The left circle diagram shows the distribution of positive and negative culture results. The right circle diagram further divides the negative results into three categories: no growth, urethral flora, and low amounts of mixed flora. A detailed breakdown of the positive samples is provided in Table 2

Of the 4295 urine samples initially included in the study, 2773 (65%) were from women and 1522 (35%) were from men. 1798 (42%) samples were from hospitalised patients and 2497 (58%) were from an outpatient setting. After excluding 290 samples (281 catheterised and 9 nephrostomy samples), 4005 midstream urine samples remained for comparative analyses of flow cytometry and culture (Fig. 1). Among these, 2458 (61%) were culture-negative, including 981 (24%) with no growth, 1142 (29%) with urethral flora, and 335 (8%) with low amounts of mixed flora (Fig. 1). Culture-positive samples accounted for 1547 samples (39%). A single species was identified in 1421 (35% of total) cultures, while 126 (3.1% of total) showed mixed flora, consisting of Gram-positive, Gram-negative, or both types of bacteria. The most frequently identified species in positive cultures were *Escherichia coli* (*E. coli*) (n = 910, 64%), *Klebsiella* spp. (n = 131, 8.5%), and *Enterococcus* spp. (n = 101, 6.5%) (Table 2).

**Table 2:** Microorganisms identified in the 1421/1547 culture positive samples

Microorganism identified	n = 1421	%
*Escherichia coli*	910	64
*Klebsiella* species	123	8.7
*Enterococcus* species	101	7.1
*Streptococcus agalactiae*	73	5.1
*Enterobacter cloacae* complex	37	2.6
*Proteus mirabilis*	23	1.6
*Staphylococcus epidermidis*	22	1.5
*Staphylococcus saprophyticus*	21	1.5
*Pseudomonas aeruginosa*	15	1.1
*Staphylococcus aureus*	14	1
*Aerococcus urinae*	9	0.6
Other Gram-positive bacteria	20	1.4
Other Gram-negative bacteria	41	2.9
Yeast	12	0.8

126 samples contained mixed flora i.e. urine samples with more than 2 types of Gram-positive bacteria, Gram-negative bacteria, or both

**Fig. 2:**
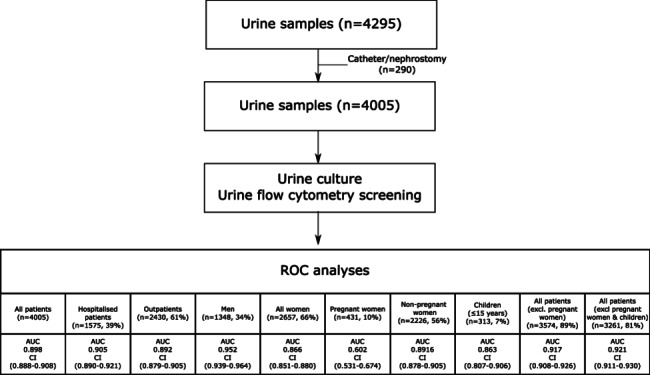
Flowchart of urine samples from patients included in the study. Receiver operating characteristic (ROC) curve analyses were performed on the total population (n = 4005) and different subpopulations

ROC curve analysis of data from the total population (n = 4005) identified bacterial counts (cells/µL) as a potential predictor of bacterial growth (AUC 0.898). However, when analysing subpopulations, the agreement between culture and urine flow cytometry was insufficient for pregnant women (n = 431, AUC 0.602) (Fig. 2). For children (n = 313, AUC 0.863), non-pregnant women (n = 2657, AUC 0.866), and outpatients (n = 2430, AUC 0.892), the agreement between the two methods was considered very good (AUC 0.8–0.9). Excellent agreement was observed in other groups, including hospitalised patients (n = 1575, AUC 0.905), men (n = 1348, AUC 0.952), and the group excluding pregnant women (n = 3574, AUC 0.917). For children (AUC 0.863), we observed a drop in sensitivity at different cut-off values for ruling out bacteriuria (80–85%). Therefore, we also performed calculations for a subpopulation excluding both pregnant women and children (n = 3261, AUC 0.921) (Fig. 2 and Supplementary Fig. S1).

We then examined different cut-off values for the flow cytometry parameter bacterial count (cells/µL) to rule out bacteriuria across all subpopulations (Tables 3 and S4). A UFC cut-off value of < 30 cells/µL resulted in a sensitivity of 96.4% and a negative predictive value (NPV) of 95.3% for the subpopulation excluding both pregnant women and children (n = 3261), leading to 1047 (32.1%) negative samples without the need for culture. At a bacterial cut-off value of 30 cells/µL, 998 samples (30.6%) were true negatives, while 49 samples (1.5%) were false negatives (negative by flow cytometry but positive by culture) (Table 3). Of these 49 false negatives, 2 contained mixed flora (3 or more bacterial types) and 2 contained yeast. In the remaining 45 samples the predominant findings were *E. coli* (34 samples), other Enterobacterales (8 samples), and *Enterococcus faecalis* (1 sample). In 3 samples, the bacterial count in culture was > 10^4^ CFU/mL (*E. coli*, *Klebsiella pneumoniae*, and *Enterococcus faecalis*), confirming these as clinically relevant false negatives (0.09%, 2 males, 1 female). The remaining 42 samples (1.3%) contained *E. coli* (34 samples) or other Enterobacterales (8 samples), with bacterial concentrations of 10^2^–10^3^ CFU/mL (7 samples) or 10^3^–10^4^ CFU/mL (35 samples) (Table 4).

**Table 3:** Predictive values of urine flow cytometry analyses to rule-out bacteriuria

Group (n)	SE^a^ (%) [95% CI]	NPV^b^ (%) [95% CI]	TN^c^	FN^d^
All (4005)	95.3 [94.2–96.3]	94.4 [93.0–95.6]	1216	72
Female (2657)	96.3 [95.0–97.3]	92.2 [89.5–94.3]	482	41
Pregnant women (431)	88.8 [80.3–94.5]	85.5 [75.0–92.8]	59	10
Women, excl. pregnant (2226)	96.9 [95.7–97.9]	93.2 [90.5–95.3]	423	31
Male (1348)	93.0 [90.3–95.2]	95.9 [94.2–97.2]	734	31
Children (313)	84.7 [75.0–91.3]	92.4 [88.2–95.4]	159	13
Outpatients (2430)	95.4 [93.9–96.6]	94.0 [92.0–95.6]	690	44
Hospitalised patients (1575)	95.3 [93.2–96.8]	94.9 [92.8–96.6]	526	28
All patients, excl. pregnant women (3574)	95.7 [94.6–96.7]	94.9 [93.5–96.0]	1157	62
All patients, excl. pregnant women and children (3261)	96.4 [95.3–97.4]	95.3 [93.9–96.5]	998	49

Predictive values of urine flow cytometry analyses compared to culture in different subpopulations to rule-out bacteriuria (UFC cut-off of < 30 cells/µl)

^a^SE, sensitivity, ^b^NPV, negative predictive value, ^c^TN, true negatives, ^d^FN, false negatives

**Table 4:** Overview of microorganisms isolated by culture from 45 urine samples classified as false negative

Microorganism identified	CFU/ml	Total number, n = 45	UTI symptom^a^, n	AST^b^
*E. coli*	10^4^–10^5^	1	–	1
*Klebsiella pneumoniae*	10^4^–10^5^	1	1	1
*Enterococcus faecalis*	10^4^–10^5^	1	–	1
*E. coli*	10^3^–10^4^	27	4	13
*E. coli*	10^2^–10^3^	7	3	5
*Citrobacter koseri*	10^3^–10^4^	1	–	–
*Klebsiella oxytoca*	10^3^–10^4^	2	–	–
*Morganella morganii*	10^3^–10^4^	1	–	–
*Proteus mirabilis*	10^3^–10^4^	2	1	2
*Pseudomonas aeruginosa*	10^3^–10^4^	1	–	1
*Pseudomonas* species	10^3^–10^4^	1	–	–

^a^UTI (urinary tract symptoms) based on information provided to the laboratory. ^b^AST = antimicrobial susceptibility testing was performed directly by the laboratory or requested by the clinician

According to European guidelines, uropathogenic growth at concentrations of 10^3^–10^4^ CFU/mL and 10^2^–10^3^ CFU/mL is relevant for reporting, and antimicrobial susceptibility testing (AST) may be required depending on the patient history [22]. Of the 42 samples, 23 were from males and 19 from females, with an average age of 66 years (range: 32–98). In 57% of these cases, the laboratory reported the clinical relevance of the finding as unclear. Although pathogens were identified and reported in all 42 samples, only half underwent AST. Among the 21 samples with AST results, testing was performed for the following reasons: 9/42 (21%) were based on patient history, 4/42 (9.5%) due to recurrent detection of the same species, and 1/42 (2.3%) at the clinician’s request. In 7/42 cases (17%), the reason for performing AST was unclear (Tables 4 and S5). As previously shown, combining bacterial counts with leukocyte counts did not improve diagnostic accuracy [4, 12, 23]. In our dataset, leukocyte counts were available for 20/42 false negative samples, with 8 of these showing elevated levels (> 10 cells/µL). Of these eight samples, five could have been identified as relevant (and prevented from being false negative) based on the patient’s clinical information, making the leukocyte values unnecessary in this context. For the remaining three samples, despite elevated leukocytes, AST was not required, as the available clinical data did not suggest the need for further investigation (Tables 4 and S5).

### Diagnostic performance of urine flow cytometry compared to urine culture to predict likely positive samples

We then examined different bacterial cut-off values to detect relevant bacteriuria across all subpopulations (Table S6). Upon analysis, we found that pregnant women had a low positive predictive value (PPV) of 62.2%, leading to their exclusion from further analysis. In contrast, the subpopulation of children exhibited a high PPV of 96.9%, suggesting that children can be included when applying rule-in criteria for bacteriuria. A bacterial cut-off value of > 4000 cells/µL to detect relevant bacteriuria resulted in 96.4% sensitivity and a 95.3% PPV for the subpopulation excluding pregnant women (n = 3574). In this group, 892 samples (25%) were considered true positives, and 40 samples (1.1%) were false positives (Table 5). The patients with false-positive samples had an average age of 61.6 years (range: 1–96). Of the false positives, 29 samples (72.5%) contained mixed flora, and 11 samples (27.5%) showed no growth in culture. Five false-positive samples were from males, and 34 from females. The most frequently identified species in the 892 screening-positive samples were *E. coli* (609, 74%), *Klebsiella* spp. (90, 11%), and *Enterobacter cloacae* complex (25, 3%) (Table 6).

**Table 5:** Predictive values of flow cytometry analyses to rule-in bacteriuria

Group (n)	SP % [95% CI]	PPV % [95% CI]	TP	FP
All (4005)	97.8 [97.1–98.4]	94.4 [92.8–95.8]	915	54
Female (2657)	96.9 [95.9–97.7]	93.2 [91.1–94.9]	657	48
Pregnant women (431)	95.9 [93.2–97.7]	62.2 [44.8–77.5]	23	14
Women, excl. pregnant (2226)	97.2 [96.1–98.1]	94.9 [93.0–96.5]	634	34
Male (1348)	99.3 [98.6–99.8]	97.7 [95.1–99.1]	258	6
Children (313)	99.6 [97.5–100.0]	96.9 [84.4–99.9]	31	1
Outpatients (2430)	98.0 [97.1–98.6]	95.0 [92.9–96.6]	569	30
Hospitalised patients (1575)	97.6 [96.4–98.4]	93.5 [90.5–95.8]	346	24
All patients, excl. pregnant women (3574)	98.1 [97.4–98.7]	95.7 [94.2–96.9]	892	40
All patients, excl. pregnant women and children (3261)	97.9 [97.2–98.5]	95.7 [94.1–96.9]	861	39

Predictive values of flow cytometry analyses compared to culture in different subpopulations to rule-in bacteriuria. (UFC cut-off Bact > 4000 cells/µl)

^a^SP, specificity, ^b^PPV, positive predictive value, ^c^TP, true positives, ^d^FP, false positives

**Table 6:** Microorganisms identified in the 822/892 UFC screening positive samples

Microorganism identified	n = 822	%
*Escherichia coli*	609	74.1
*Klebsiella* species	90	10.9
*Enterobacter cloacae* complex	25	3.0
*Enterococcus* species	19	2.3
Other Gram-negative bacteria	18	2.2
Other Gram-positive bacteria	16	1.9
*Proteus mirabilis*	11	1.3
*Pseudomonas aeruginosa*	10	1.2
*Aerococcus urinae*	7	0.9
*Staphylococcus aureus*	6	0.7
*Staphylococcus epidermidis*	4	0.5
*Streptococcus agalactiae*	3	0.4
*Staphylococcus saprophyticus*	2	0.2
Yeast	2	0.2

70 samples contained mixed flora i.e. urine samples with more than 2 types of Gram-positive bacteria, Gram-negative bacteria, or both

In the subpopulation excluding pregnant women (n = 3574), the Bact Info-flag “Gram Neg” was activated in 543 (61%) of 892 samples with bacterial counts > 4000/µL, considered positive in screening. All flagged samples were culture positive. Except for two cases, all samples flagged as “Gram Neg?” exhibited at least one Gram-negative bacterial species in culture. There was full agreement between the Gram classification of the “Gram Neg?” flag in 523 samples (96%), partial agreement in 18 samples (3.3%), and disagreement in 2 samples (0.37%) (Table 7).

**Table 7:** Culture results from samples flagged with Bact Info flag “Gram NEG?” by UFC

Bacterial classification from urine culture identification	UF-5000 Bact Info flag “Gram NEG?”
Gram negatives	523
Gram positives	2
Mixed flora (Gram-negatives and Gram-positives)	18
Culture negative (no growth or < 10^**4**^ CFU/mL)	0
Total	543

**Fig. 3:**
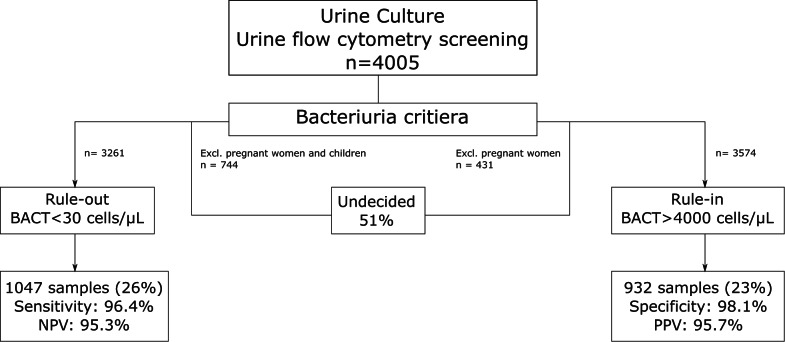
Flowchart of the theoretical performance of the developed bacteriuria algorithm. *NPV* negative predictive value, *PPV* positive predictive value

In conclusion, we here propose an algorithm for sorting urine samples based on screening results, similar to the approach of Gehringer et al. [14] (Fig. 3). To rule out relevant bacteriuria, a cut-off of < 30 cells/µL was selected based on sensitivity and negative predictive value (NPV) of > 95%. For this criterion, we recommend excluding two subpopulations—pregnant women and children—due to their low sensitivity and NPV. In the subgroup excluding these patients (n = 3261), this approach resulted in that 32% of all samples (55% of all negative samples) could be reported as negative without culture. To rule in relevant bacteriuria, a cut-off of > 4000 cells/µL was chosen. Pregnant women should be excluded from this analysis due to a low positive predictive value (PPV). Among the 2058 samples in the “undecided group” with bacterial counts between 30 and 4000 cells/µl (51% of all samples), 604 (29%) were culture positive and 1454 (71%) were culture negative. This intermediate group represents a substantial proportion of tested samples and includes both clinically relevant positives and true negatives, highlighting the need for continued urine culture in this group (Fig. 3). The detailed performance of the algorithm when applied to the relevant subpopulations is summarised in Table 8 and the reduction in culture is visualised in Supplementary Fig. S2.

**Table 8:** Performance of the developed algorithm to exclude or detect bacteriuria

	All n = 4005	Female n = 2657	Male n = 1348	Outpatients n = 2430	Hospitalised patients n = 1575	All patients excl. pregnant women n = 3574	All patients excl. pregnant women and children n = 3261
*Rule-out (*< *30 cells/µl)*							
SE^a^, %	95.5	96.3	93.0	95.4	95.3	95.7	96.4
NPV^b^, %	94.4	92.2	95.9	94.0	94.9	94.9	95.3
*Rule-in (*> *4000 cells/µl)*							
SP^c^, %	97.8	96.9	99.3	98.0	97.6	98.1	97.9
PPV^d^, %	94.4	93.2	97.7	95.0	93.5	95.7	95.7
Number of undecided samples, i.e. ≥ 30 and ≤ 4000 cells/µl (% of total within that group)	1748 (43.6)	1430 (53.8)	1029 (23.7)	1097 (45.1)	1575 (41.3)	1423 (39.8)	1314 (40.3)
Reduction of number negative cultures n (%)	1288 (32.2)	523 (19.7)	765 (56.8)	734 (30.2)	554 (35.2)	1219 (34.1)	1047 (32.1)

For the reduction in number of negative cultures for all samples and across different relevant patient subpopulations see also Supplementary Fig. S2

^a^SE, sensitivity, ^b^NPV, negative predictive value, ^c^SP, specificity, ^d^PPV, positive predictive value

## Discussion

Urinary tract infections (UTIs) are among the most common bacterial infections, and while urine culture remains a frequently used diagnostic method in clinical microbiology laboratories, there is a growing need for rapid and accurate testing to ensure timely treatment [24]. This study evaluated the performance of urine flow cytometric screening using the UF-5000 and proposed an optimised workflow to efficiently differentiate between negative and likely positive urine samples while maintaining high diagnostic accuracy.

We analysed 4005 samples, comparing UFC results with culture results. Diagnostic cut-offs vary across laboratories due to differences in patient populations and different definitions used to classify significant bacteriuria. In our study, a UFC cut-off of < 30 cells/µl provided a balance between high sensitivity and reduced culture workload. Introduction of this method would decrease the need for urine cultures by > 30%, which is consistent with previous studies [12, 23, 25]. This reduction has substantial benefits, including lower costs, improved turnaround times, and decreased laboratory workload. Faster reporting of negative results allows > 30% of patients to receive same-day results. The rapid turnaround benefits both clinicians and patients by facilitating timely decision-making and improving antimicrobial stewardship. Despite these advantages, certain patient groups posed challenges. We observed lower agreement between flow cytometry and culture results in pregnant women, necessitating their exclusion from this workflow. Similarly, children showed reduced sensitivity and negative predictive value (NPV), leading to their exclusion as well. This aligns with Swedish UTI guidelines, which emphasise different diagnostic approaches for children and pregnant women [15]. Pregnant women are particularly at risk of complications, such as preterm birth and maternal hypertension, when bacteriuria remains untreated. Therefore, their urine samples should be cultured to detect uropathogens and group B streptococci [26]. Our data reinforce the recommendation that urine culture should remain the gold standard for these populations. Minimising false negatives is critical, as missing relevant infections can delay treatment.

False negatives are a known limitation of any screening approach. While false negatives do occur, they can be reduced through careful interpretation of the results and consideration of clinical factors. In our study, a total of 49 samples (1.5%) were negative by flow cytometry but positive by culture, of which three samples (0.09%) showed clinically significant bacteriuria (≥ 10^4^ CFU/mL), representing the most relevant false negatives in our cohort (Tables 4 and S5). The remaining 46 samples either contained yeast (2/46), mixed flora (2/46) or had low bacterial counts (10^2^–10^4^ CFU/mL) (42/46). This highlights that most false negatives in urine screening involve low concentration uropathogens, which are more challenging to detect but may still be clinically relevant. Importantly, clinical context plays a critical role in interpretation. Our findings further highlight the importance of clear clinical communication. In many cases with low bacterial concentrations (10^2^–10^4^ CFU/mL), the absence of detailed clinical information led to uncertainty regarding the clinical significance and the need for AST. In 57% of these FN samples, the laboratory noted the clinical relevance as unclear, and in some cases, the indication for AST was not evident. This supports the need for improved dialogue between clinicians and the microbiology laboratory to ensure accurate interpretation and appropriate testing decisions, particularly in borderline or diagnostically complex cases. When available patient symptoms were taken into account (if provided to the laboratory), the number of potential false negatives dropped from 42 to 29 (0.89%), illustrating how combining clinical and laboratory information enhances diagnostic safety. This highlights the importance of clinicians providing accurate clinical information to support laboratory decision-making. Nevertheless, in specific high-risk populations, such as immunocompromised patients and those in intensive care, urine culture remains indispensable, even if screening results are negative. These groups may present with lower bacterial loads that are clinically important but harder to detect through screening alone. Therefore, while UFC offers substantial benefits in reducing unnecessary cultures and streamlining diagnostics, careful consideration of patient population and clinical presentation remains essential to avoid underdiagnosis. Tailored sensitivity thresholds or complementary testing may be appropriate in high-risk settings to ensure optimal patient care [27, 28]. To further reduce the risk of false negatives, especially in samples with low bacterial counts, laboratories may consider implementing a structured decision-making protocol that combines flow cytometry results with relevant available clinical information such as symptoms, history of recurrent infections, or other risk factors. Such a workflow can guide targeted antimicrobial susceptibility testing (AST) in borderline cases, ensuring that clinically relevant infections are not missed. This approach emphasises the importance of ongoing communication between clinicians and laboratory personnel to enhance diagnostic accuracy and patient care.

While elevated leukocyte counts are commonly associated with infection, our findings confirm that such parameters must be interpreted in the context of clinical information. In several cases (3), elevated leukocytes were observed in samples for which no AST was performed, as the clinical data did not indicate the need for further workup. This illustrates the inherent limitation of using UFC as a standalone tool and reinforces the essential role of clinical context in guiding appropriate diagnostics. A reliable screening algorithm must therefore be supported by accurate and accessible clinical information to optimise diagnostic decision-making. Each laboratory must carefully assess the risks and benefits of screening methods compared to urine culture to ensure appropriate diagnostic decisions.

UFC has long been recognised as an effective tool for identifying bacteriuria [13]. However, its predictive performance varies across patient subpopulations. Our study found that the positive predictive value (PPV) for pregnant women was only 62.2%, whereas in children, it was notably high at 96.9%, suggesting that flow cytometry could be useful for paediatric patients as previously shown [29]. In the subgroup analysis excluding pregnant women (n = 3574), a bacterial cut-off value of > 4000 cells/µl demonstrated strong diagnostic performance (96.4% sensitivity, 95.3% PPV). This is in line with previous studies and highlights the methods’ potential to accurately identify relevant bacteriuria across a broad patient population [4, 9, 14]. The 40 (1.1%) false positive samples were mostly due to mixed flora, indicating contamination or polymicrobial infections that are difficult to distinguish using flow cytometry alone. Given that all screening-positive samples proceed to culture, these cases can be clarified through confirmatory urine culture, ensuring accurate reporting and minimising the risk of misclassification.

Identification of the UTI-causing bacteria as soon as possible is important, especially in cases of UTI complicated by bacteraemia or sepsis, where targeted therapy could improve patient outcomes. In this study, we evaluated the performance of the Bact Info-flag “Gram Neg?” in a subpopulation excluding pregnant women. Our findings demonstrate that the flag was activated in 61% of samples classified as positive based on bacterial count (> 4000/µl). Notably, all flagged samples were culture-positive, underscoring the high specificity of this indicator for detecting true bacterial infections. A key observation was the strong concordance between the Gram classification provided by the flag and the culture results. In 96% of cases, there was full agreement between the flag and the Gram-negative species identified in culture. Additionally, partial agreement, where both Gram-negative and Gram-positive bacteria were detected, occurred in 3.3% of samples. Discrepancies were observed in only two cases (0.37%), suggesting a minimal rate of misclassification. The high specificity of the “Gram Neg?” flag highlights its potential use in laboratory workflows, particularly in rapidly distinguishing Gram-negative infections. This aligns with previous findings, where the “Gram Neg?” flag demonstrated good sensitivity and optimal specificity for predicting Gram-negative bacteria in culture, with an overall agreement of 99.8% when Gram negatives were present alone or together with Gram positives, and a very low discordance rate of 0.2% [4]. Given that Gram-negative bacteria often are associated with more severe UTIs and may require specific antibiotic treatments, early identification can support timely clinical decision-making. In urgent cases where immediate treatment is necessary, the ‘Gram Neg?’ flag may support early, targeted antibiotic initiation prior to culture confirmation. This could be particularly valuable in emergency settings or for vulnerable patient groups where treatment delays may have serious consequences. While further validation is needed, the flag may serve as a useful adjunct to guide empiric therapy decisions in appropriate clinical contexts. This could be particularly valuable in emergency settings or for vulnerable patient groups where treatment delays may have serious consequences. While further validation is needed, the flag may serve as a useful adjunct to guide empiric therapy decisions in appropriate clinical contexts.

Our proposed workflow for sorting urine samples based on screening results aims to optimise the laboratory workflow by reducing unnecessary cultures while maintaining diagnostic accuracy. The application of a < 30 BACT/µl rule for negative samples led to a 32% reduction in cultures, with a 55% decrease among negative samples in a population were pregnant women and children are excluded. This supports previous findings where a similar strategy was proposed highlighting the potential of such an approach in reducing laboratory workload while maintaining diagnostic safety [14]. The ability to confidently exclude a significant proportion of negative samples without additional testing is crucial for improving laboratory efficiency and resource allocation. For ruling in relevant bacteriuria, we established a cut-off of > 4000 cells/µl, providing a reliable indicator of clinically significant bacterial presence. The intermediate group with bacterial counts between 30 and 4000 cells/µl (51%) represents a diagnostic grey zone where culture remains necessary. Clinical context and additional diagnostic information are essential for safe decision-making until further refinements or supporting tools become available. The overall performance of our algorithm, as summarised in Table 8, supports its feasibility in routine laboratory practice, both for in- and out-patient samples. By implementing a structured decision-making process based on screening results, we can streamline urine diagnostics, minimising unnecessary cultures while ensuring that clinically significant cases are properly identified. Future studies should further validate these cut-offs in larger and more diverse populations, particularly in settings with different patient demographics or clinical guidelines. Additionally, integrating this algorithm with automated reporting systems could further enhance its practical application in high-throughput laboratories. Carryover and cross-contamination were minimal, which is crucial for microbiology screening, as the same tube is used for urine culture when the screening result is positive.

Limitations of the study: This study has several limitations. Its retrospective design may introduce selection bias and limits the control over sample handling and data collection. According to the manufacturer’s protocol, Sysmex UF-5000 analysis should ideally be performed on fresh urine samples within 4 h without preservatives. However, some outpatient samples required longer transport times and these could not be separately identified or excluded. This may affect the generalisability of our results to other settings where sample transport and processing times differ. Additionally, diagnostic cut-offs and bacteriological significance thresholds vary between laboratories and countries, which may limit direct applicability of our proposed cut-offs beyond our local context. European guidelines also highlight the importance of considering local epidemiology and clinical context when implementing new screening protocols [16, 22]. Future prospective studies in diverse clinical settings would help confirm the robustness and transferability of our findings.

## Conclusions

Our proposed algorithm for sorting urine samples based on flow cytometry screening results aims to optimise laboratory workflow by reducing unnecessary cultures while maintaining diagnostic accuracy. Using a bacterial cut-off of < 30 cells/µl (AUC 0.921) to help identifying negative samples led to a 32% reduction in cultures overall, and a 55% reduction of negative samples when excluding pregnant women and children. For ruling in clinically relevant bacteriuria, a cut-off of > 4000 cells/µl was established when pregnant women are excluded (AUC 0.917), enabling early identification of potential bacteriuria and offering the opportunity to initiate treatment before culture confirmation. Samples with bacterial counts between 30 and 4000 cells/µl represent a diagnostic grey zone where culture remains necessary. This approach balances efficiency and safety, supporting previous findings on workload reduction while maintaining high sensitivity. Further validation in diverse populations is warranted. Implementation of this algorithm can enhance routine urine diagnostics by streamlining decision-making and improving resource allocation.

## Electronic supplementary material

Below is the link to the electronic supplementary material.

Supplementary Material 1.

Supplementary Material 2.

Supplementary Material 3.

## Data Availability

The datasets used and analysed during the current study are available from the corresponding author upon reasonable request.

## Abbreviations

AST: Antimicrobial susceptibility testing
AUC: Area under the curve
CRAN: Comprehensive R archive network, a repository of R packages and software
*E. coli*: *Escherichia coli*
FN: False negatives
FP: False positives
NPV: Negative predictive value
PPV: Positive predictive value
ROC: Receiver operating characteristic
SE: Sensitivity
SP: Specificity
TN: True negatives
TP: True positives
UFC: Urine flow cytometry
UTI: Urinary tract infection
