# Supplementary figures and images for: Flow cytometry for screening and prioritisation of urine samples: a retrospective comparison with culture
Source: BMC Infect Dis. 2025 Jul 30;25:960. doi: 10.1186/s12879-025-11374-8 (PMC12312330; doi:10.1186/s12879-025-11374-8)

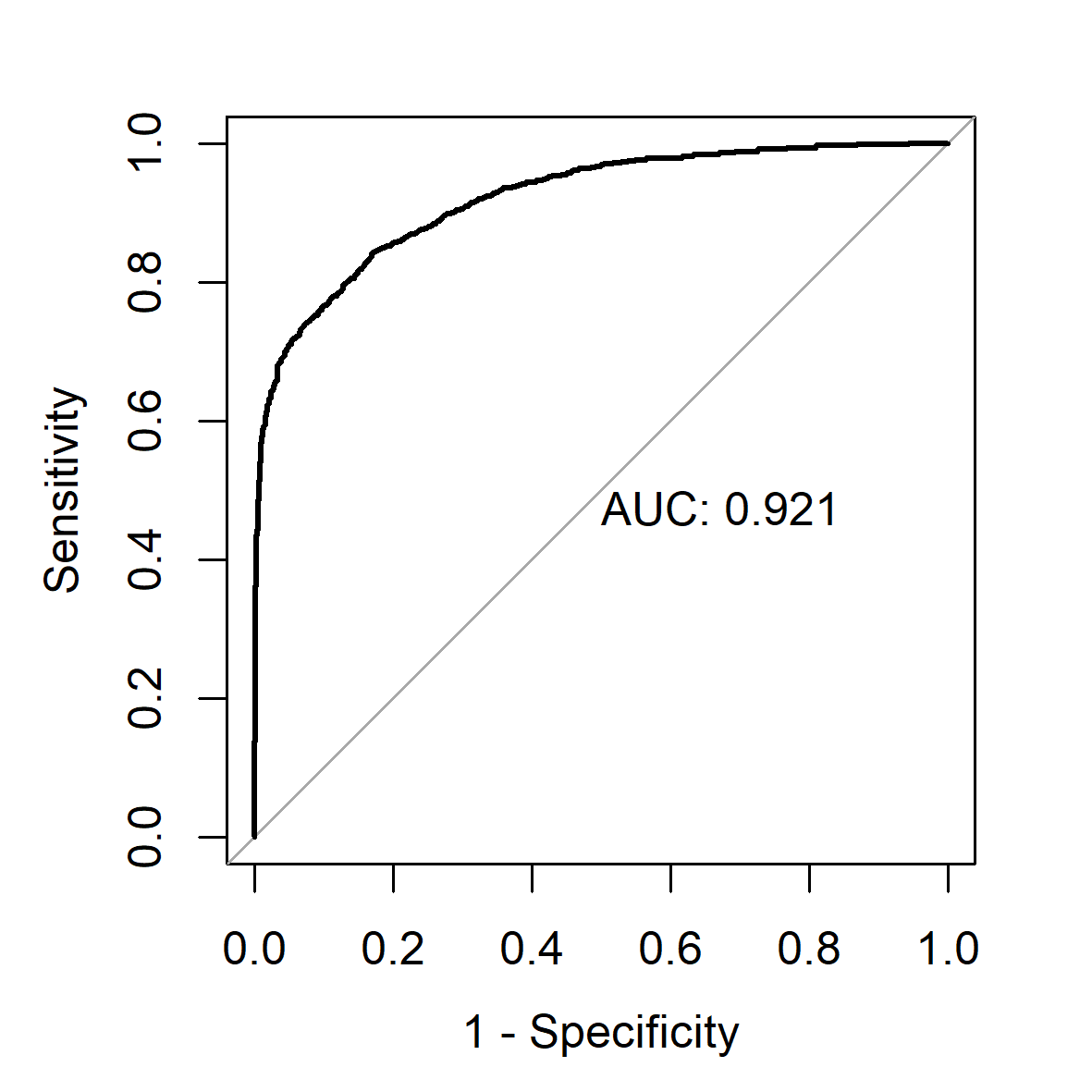

Supplement: Supplementary file 1 — Supplementary Material 1. [file 12879_2025_11374_MOESM1_ESM.tiff]

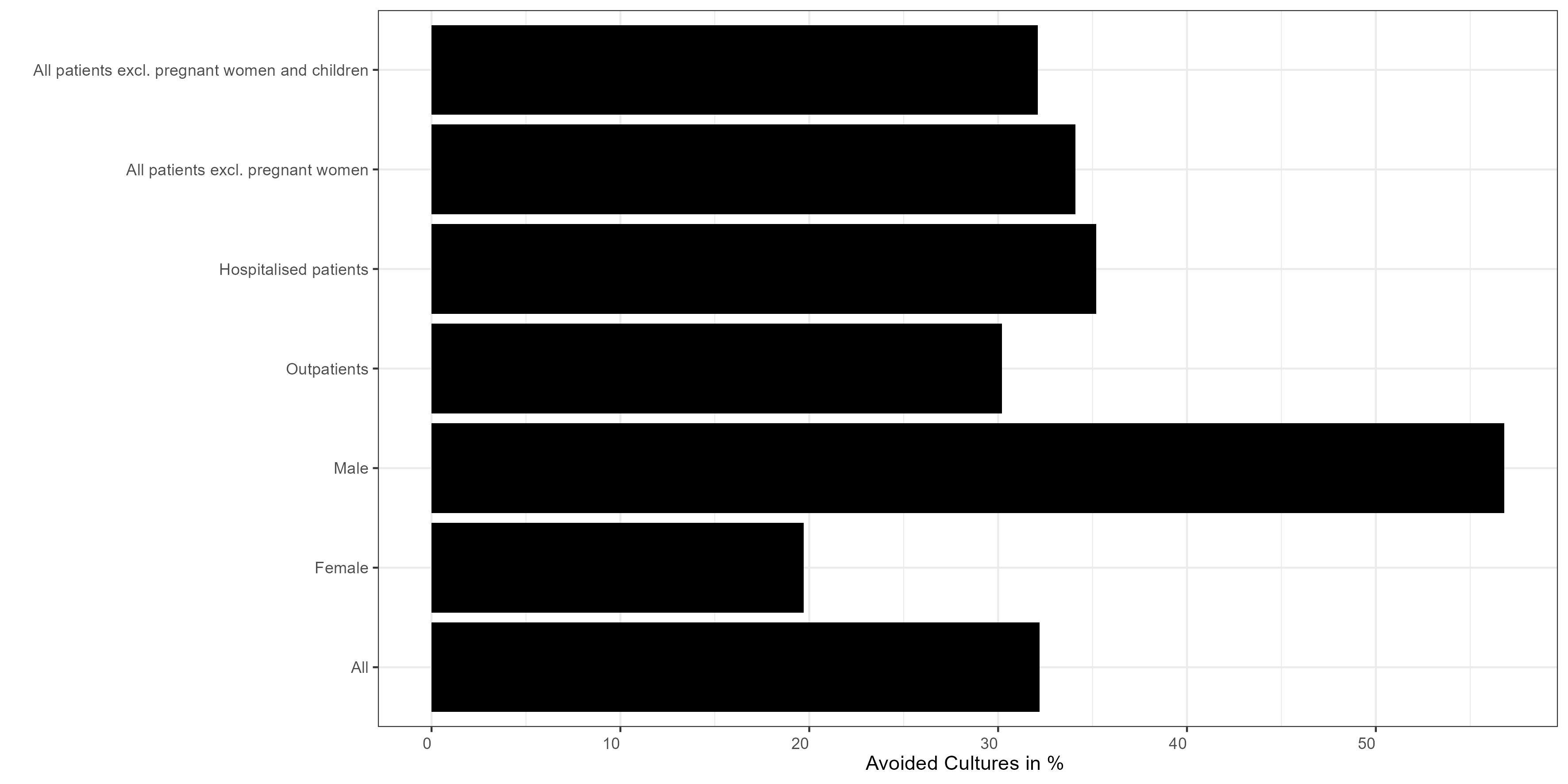

Supplement: Supplementary file 2 — Supplementary Material 2. [file 12879_2025_11374_MOESM2_ESM.tiff]
