# Supplementary material for: Flow cytometry for screening and prioritisation of urine samples: a retrospective comparison with culture
Source: BMC Infect Dis. 2025 Jul 30;25:960. doi: 10.1186/s12879-025-11374-8 (PMC12312330; doi:10.1186/s12879-025-11374-8)
Supplement: Supplementary file 3 — Supplementary Material 3. [file 12879_2025_11374_MOESM3_ESM.docx]

Supplementary material

Supplementary figure legends

Figure S1. Representative ROC curve of the patient subgroup ”All patients, excl. pregnant women and children” (n=3261, AUC 0.921)

Figure S2. Percentage reduction of negative cultures across different.

Supplementary Tables

Table S1. Number of rinses based on bacterial count in UF-5000.

| Bacterial count  (cells/µl) | Rinse mode – no. of rinses | | | | | |
| --- | --- | --- | --- | --- | --- | --- |
|  | 1-2-3 | 1-3-4 | 2-2-3 | 2-3-3 | 2-4-4 | 2-5-5 |
| 1x10^6^-9.9x10^6^  1x10^7^-9.9x10^7^  ≥ 10^8^ | 1  2  3 | 1  3  4 | 2  2  3 | 2  3  3 | 2  4  4 | 2  5  5 |

Table S2. Results from carryover analyses at six different rinse modes in the Sysmex UF-5000 flow cytometer.

|  | Carryover (UFC BACT cells/ml) | | | | | |
| --- | --- | --- | --- | --- | --- | --- |
| Rinse mode | Patient no. | Sample type. bacterial isolate | Sysmex run 1 | Sysmex run 2 | Sysmex run 3 | Carryover rate (%) |
| 1-2-3 | 1 | Urine, *Klebsiella pneumoniae* | 3.95x10^7^ | 3.55x10^7^ | 5.37x10^7^ | 0.002 |
|  |  | Sterile saline | 1.10x10^3^ | 2.30x10^3^ | 0.0 |  |
|  | 2 | Urine, *Escherichia coli* | 1.00x10^8^ | 1.00x10^8^ | 1.00x10^8^ | 0.001 |
|  |  | Sterile saline | 1.10x10^3^ | 2.30x10^3^ | 0.0 |  |
|  | 3 | Urine, *Escherichia coli* | 9.80x10^7^ | 9.80x10^7^ | 9.92x10^7^ | 0.001 |
|  |  | Sterile saline | 3.50x10^3^ | 3.50x10^3^ | 2.30 x10^3^ |  |
|  | 4 | Urine, *Citrobacter koseri* | 8.09x10^6^ | 8.11x10^6^ | 8.09x10^6^ | 0.000 |
|  |  | Sterile saline | 0.00 | 0.00 | 0.00 |  |
|  | 5 | Urine, *Citrobacter freundii* | 2.45x10^7^ | 2.18x10^7^ | 2.18x10^7^ | 0.000 |
|  |  | Sterile saline | 2.30 x10^3^ | 1.10x10^3^ | 2.30 x10^3^ |  |
|  | 6 | Urine, *Escherichia coli* | 3.25x10^7^ | 3.17x10^7^ | 3.22x10^7^ | 0.003 |
|  |  | Sterile saline | 1.10x10^3^ | 2.30x10^3^ | 0.00 |  |
|  | 7 | Urine, *Klebsiella aerogenes* | 6.03x10^7^ | 5.25x10^7^ | 4.91x10^7^ | 0.000 |
|  |  | Sterile saline | 2.30x10^3^ | 1.10x10^3^ | 2.30x10^3^ |  |
| 1-3-4 | 8 | Urine, *Citrobacter koseri* | 7.95x10^6^ | 8.28x10^6^ | 9.31x10^6^ | 0.025 |
|  |  | Sterile saline | 2.30 x10^3^ | 0.00 | 0.00 |  |
|  | 9 | Urine, *Escherichia coli* | 2.21x10^6^ | 2.29x10^6^ | 2.54x10^6^ | 0.043 |
|  |  | Sterile saline | 1.10x10^3^ | 0.00 | 0.00 |  |
|  | 10 | Urine, Urethral flora | 4.97x10^6^ | 5.01x10^6^ | 5.74x10^6^ | 0.000 |
|  |  |  | 2.30x10^3^ | 1.10x10^3^ | 2.30x10^3^ |  |
|  | 11 | Urine, *Escherichia coli* | 7.34x10^7^ | 7.57x10^7^ | 7.73x10^7^ | 0.001 |
|  |  | Sterile saline | 1.10x10^3^ | 1.10x10^3^ | 0.00 |  |
|  | 12 | Urine, *Escherichia coli* | 8.16x10^7^ | 8.32x10^7^ | 8.49x10^7^ | 0.003 |
|  |  | Sterile saline | 2.30x10^3^ | 0.00 | 0.00 |  |
|  | 13 | Urine, *Escherichia coli* | 9.42 x10^7^ | 9.68 x10^7^ | 9.27 x10^7^ | 0.000 |
|  |  | Sterile saline | 0.00 | 0.00 | 0.00 |  |
|  | 14 | Urine, *Klebsiella pneumoniae* | 9.96 x10^7^ | 9.77 x10^7^ | 1.00 x10^8^ | 0.001 |
|  |  | Sterile saline | 1.10x10^3^ | 0.00 | 0.00 |  |
| 2-2-3 | 8 | Urine, *Citrobacter koseri* | 1.06x10^7^ | 1.07x10^7^ | 1.11x10^7^ | 0.021 |
|  |  | Sterile saline | 2.30 x10^3^ | 0.00 | 0.00 |  |
|  | 9 | Urine, *Escherichia coli* | 2.18x10^6^ | 2.01x10^6^ | 2.17x10^6^ | 0.000 |
|  |  | Sterile saline | 0.00 | 1.10x10^3^ | 0.00 |  |
|  | 11 | Urine, *Escherichia coli* | 7.63x10^7^ | 7.71x10^7^ | 7.76x10^7^ | -0.003 |
|  |  | Sterile saline | 1.10x10^3^ | 1.10x10^3^ | 3.50x10^3^ |  |
|  | 13 | Urine, *Escherichia coli* | 9.82x10^7^ | 9.97x10^7^ | 9.42x10^7^ | -0.003 |
|  |  | Sterile saline | 1.10x10^3^ | 2.30x10^3^ | 3.50x10^3^ |  |
|  | 14 | Urine, *Klebsiella pneumoniae* | 1.00 x10^8^ | 1.00 x10^8^ | 1.00 x10^8^ | 0.000 |
|  |  | Sterile saline | 0.00 | 0.00 | 0.00 |  |
| 2-3-3 | 1 | Urine, *Klebsiella pneumoniae* | 3.27x10^7^ | 3.43 x10^7^ | 3.50 x10^7^ | 0.003 |
|  |  | Sterile saline | 1.10x10^3^ | 0.00 | 0.00 |  |
|  | 2 | Urine, *Escherichia coli* | 1.00 x10^8^ | 1.00 x10^8^ | 1.00 x10^8^ | 0.003 |
|  |  | Sterile saline | 7.10x10^3^ | 0.00 | 4.60x10^3^ |  |
|  | 4 | Urine, *Citrobacter koseri* | 8.98 x10^6^ | 8.68 x10^6^ | 8.78 x10^6^ | 0.000 |
|  |  | Sterile saline | 0.00 | 2.30x10^3^ | 0.00 |  |
|  | 5 | Urine, *Citrobacter freundii* | 2.19x10^7^ | 2.35x10^7^ | 2.30 x10^7^ | 0.005 |
|  |  | Sterile saline | 1.10x10^3^ | 0.00 | 0.00 |  |
|  | 6 | Urine, *Escherichia coli* | 3.52x10^7^ | 3.38x10^7^ | 3.30 x10^7^ | 0.000 |
|  |  | Sterile saline | 0.00 | 0.00 | 0.00 |  |
|  | 7 | Urine, *Klebsiella aerogenes* | 6.01x10^7^ | 5.69x10^7^ | 6.59 x10^7^ | 0.000 |
|  |  | Sterile saline | 0.00 | 0.00 | 0.00 |  |
| 2-4-4 | 15 | Urine, *Enterobacter cloacae* | 2.61x10^7^ | 2.40x10^7^ | 2.53x10^7^ | 0.000 |
|  |  | Sterile saline | 0.00 | 0.00 | 0.00 |  |
|  | 16 | Urine, *Enterobacter cloacae* | 5.63x10^7^ | 5.57x10^7^ | 5.60x10^7^ | 0.000 |
|  |  | Sterile saline | 0.00 | 0.00 | 0.00 |  |
|  | 17 | Urine, *Citrobacter koseri* | 9.97x10^6^ | 1.07x10^7^ | 1.13x10^7^ | 0.000 |
|  |  | Sterile saline | 0.00 | 1.10x10^3^ | 0.00 |  |
|  | 18 | Urine, *Enterobacter cloacae* | 7.14x10^7^ | 6.84x10^7^ | 7.19x10^7^ | 0.000 |
|  |  | Sterile saline | 2.30x10^3^ | 0.00 | 2.30x10^3^ |  |
|  | 19 | Urine, *Citrobacter koseri* | 1.00 x10^8^ | 1.00 x10^8^ | 1.00 x10^8^ | 0.001 |
|  |  | Sterile saline | 1.10x10^3^ | 2.30x10^3^ | 0.00 |  |
| 2-5-5 | 20 | Urine, *Proteus mirabilis* | 1.63x10^6^ | 1.49x10^6^ | 1.67x10^6^ | 0.000 |
|  |  | Sterile saline | 0.00 | 0.00 | 0.00 |  |
|  | 21 | Urine, *Citrobacter koseri* | 6.68x10^6^ | 7.18x10^6^ | 7.47x10^6^ | 0.000 |
|  |  | Sterile saline | 0.00 | 0.00 | 0.00 |  |
|  | 22 | Urine, *Citrobacter koseri* | 6.34x10^6^ | 6.49x10^6^ | 5.97x10^6^ | 0.000 |
|  |  | Sterile saline | 0.00 | 0.00 | 0.00 |  |
|  | 23 | Urine, *Klebsiella pneumoniae* | 2.29x10^7^ | 2.19x10^7^ | 2.31x10^7^ | 0.000 |
|  |  | Sterile saline | 0.00 | 0.00 | 0.00 |  |
|  | 24 | Urine, *Klebsiella oxytoca* | 3.68x10^7^ | 3.85x10^7^ | 3.76x10^7^ | 0.003 |
|  |  | Sterile saline | 1.10x10^3^ | 0.00 | 0.00 |  |
|  | 25 | Urine, *Escherichia coli* | 9.83x10^7^ | 9.81x10^7^ | 9.64x10^7^ | 0.000 |
|  |  | Sterile saline | 0.00 | 0.00 | 0.00 |  |

Urine samples used here contained >10^5^CFU/ml according to standard microbiological methodology.

Table S3. Results from cross-contamination analyses using 25 different urine samples (>10^5^CFU/ml).

| **Growth in triplicate saline samples (CFU/ml)** | | | |
| --- | --- | --- | --- |
| **Bacterial isolate in urine sample. >10^5^CFU/ml** | **Sample 1** | **Sample 2** | **Sample 3** |
| *Citrobacter freundii* | 0 | 0 | 0 |
| *Citrobacter koseri* | 0 | 0 | 0 |
| *Citrobacter koseri* | 0 | 0 | 0 |
| *Citrobacter koseri* | 0 | 0 | 0 |
| *Citrobacter koseri* | 0 | 0 | 0 |
| *Citrobacter koseri* | 0 | 0 | 0 |
| *Citrobacter koseri* | 0 | 0 | 0 |
| *Enterobacter cloacae* | 0 | 0 | 0 |
| *Enterobacter cloacae* | 0 | 0 | 0 |
| *Enterobacter cloacae* | 0 | 0 | 0 |
| *Escherichia coli* | 0 | 0 | 0 |
| *Escherichia coli* | 0 | 0 | 0 |
| *Escherichia coli* | 0 | 0 | 0 |
| *Escherichia coli* | 0 | 0 | 0 |
| *Escherichia coli* | 1 CFU | 0 | 1 CFU |
| *Escherichia coli* | 0 | 0 | 0 |
| *Escherichia coli* | 0 | 0 | 0 |
| *Escherichia coli* | 0 | 0 | 0 |
| *Escherichia coli* | 0 | 0 | 0 |
| *Klebsiella aerogenes* | 0 | 0 | 0 |
| *Klebsiella oxytoca* | 0 | 0 | 0 |
| *Klebsiella pneumoniae* | 0 | 0 | 0 |
| *Klebsiella pneumoniae* | 0 | 0 | 0 |
| *Klebsiella pneumoniae* | 0 | 0 | 0 |
| *Proteus mirabilis* | 0 | 0 | 0 |

Data show growth in triplicate saline samples after each urine sample in the Sysmex UF-5000 flow cytometer.

Table S4. Predictive values of UFC compared to culture to exclude bacteriuria.

| **FCA cut-off** | **Group. n** | **SE^a^ %** | **NPV^b^ %** | **TP^c^** | **TN^d^** | **FP^e^** | **FN^f^** |
| --- | --- | --- | --- | --- | --- | --- | --- |
| 10 | All patients, 4005 | 98.0 | 96.3 | 1516 | 817 | 1641 | 31 |
| 20 |  | 96.4 | 95.0 | 1492 | 1041 | 1417 | 55 |
| 25 |  | 95.7 | 94.5 | 1481 | 1139 | 1319 | 66 |
| 30 |  | 95.3 | 94.4 | 1475 | 1216 | 1242 | 72 |
| 40 |  | 93.9 | 93.3 | 1453 | 1303 | 1155 | 94 |
| 10 | All women, 2657 | 98.9 | 95.5 | 1090 | 254 | 1301 | 12 |
| 20 |  | 97.3 | 92.5 | 1072 | 370 | 1185 | 30 |
| 25 |  | 96.6 | 91.8 | 1064 | 428 | 1127 | 38 |
| 30 |  | 96.3 | 92.2 | 1061 | 482 | 1073 | 41 |
| 40 |  | 94.9 | 90.6 | 1046 | 539 | 1016 | 56 |
| 10 | Pregnant women, 431 | 98.9 | 96.3 | 88 | 26 | 316 | 1 |
| 20 |  | 92.1 | 84.1 | 82 | 37 | 305 | 7 |
| 25 |  | 91.0 | 84.9 | 81 | 45 | 297 | 8 |
| 30 |  | 88.8 | 85.5 | 79 | 59 | 283 | 10 |
| 40 |  | 83.1 | 81.9 | 74 | 68 | 274 | 15 |
| 10 | Women, (excl. pregnant excl.), 2226 | 98.9 | 95.4 | 1002 | 228 | 985 | 11 |
| 20 |  | 97.7 | 93.5 | 990 | 333 | 880 | 23 |
| 25 |  | 97.0 | 92.7 | 983 | 383 | 830 | 30 |
| 30 |  | 96.9 | 93.2 | 982 | 423 | 790 | 31 |
| 40 |  | 96.0 | 92.0 | 972 | 471 | 742 | 41 |
| 10 | Men, 1348 | 95.7 | 96.7 | 426 | 563 | 340 | 19 |
| 20 |  | 94.4 | 96.4 | 420 | 671 | 232 | 25 |
| 25 |  | 93.7 | 96.2 | 417 | 711 | 192 | 28 |
| 30 |  | 93.0 | 95.9 | 414 | 734 | 169 | 31 |
| 40 |  | 91.5 | 95.3 | 407 | 764 | 139 | 38 |
| 10 | Children (≤15 years), 313 | 94.1 | 95.1 | 80 | 97 | 131 | 5 |
| 20 |  | 84.7 | 91.0 | 72 | 131 | 97 | 13 |
| 25 |  | 84.7 | 91.9 | 72 | 148 | 80 | 13 |
| 30 |  | 84.7 | 92.4 | 72 | 159 | 69 | 13 |
| 40 |  | 82.4 | 91.9 | 70 | 171 | 57 | 15 |
| 10 | Outpatients, 2430 | 98.2 | 96.5 | 940 | 468 | 1005 | 17 |
| 20 |  | 96.7 | 94.9 | 925 | 596 | 877 | 32 |
| 25 |  | 95.8 | 94.2 | 917 | 651 | 822 | 40 |
| 30 |  | 95.4 | 94.0 | 913 | 690 | 783 | 44 |
| 40 |  | 93.8 | 92.6 | 898 | 742 | 731 | 59 |
| 10 | Hospitalised patients, 1575 | 97.6 | 96.1 | 576 | 349 | 636 | 14 |
| 20 |  | 96.1 | 95.1 | 567 | 445 | 540 | 23 |
| 25 |  | 95.6 | 94.9 | 564 | 488 | 497 | 26 |
| 30 |  | 95.3 | 94.9 | 562 | 526 | 459 | 28 |
| 40 |  | 94.1 | 94.1 | 555 | 561 | 424 | 35 |
| 10 | All patients excl. pregnant women, 3574 | 97.9 | 96.3 | 1428 | 791 | 1325 | 30 |
| 20 |  | 96.7 | 95.4 | 1410 | 1004 | 1112 | 48 |
| 25 |  | 96.0 | 95.0 | 1400 | 1094 | 1022 | 58 |
| 30 |  | 95.7 | 94.9 | 1396 | 1157 | 959 | 62 |
| 40 |  | 94.6 | 94.0 | 1379 | 1235 | 881 | 79 |
| 10 | All patients excl. pregnant women + children, 3261 | 98.2 | 96.5 | 1348 | 694 | 1194 | 25 |
| 20 |  | 97.5 | 96.1 | 1338 | 873 | 1015 | 35 |
| 25 |  | 96.7 | 95.5 | 1328 | 946 | 942 | 45 |
| 30 |  | 96.4 | 95.3 | 1324 | 998 | 890 | 49 |
| 40 |  | 95.3 | 94.3 | 1309 | 1064 | 824 | 64 |

^a^SE. sensitivity. ^b^NPV. negative predictive value. ^c^TP. true positives. ^d^TN. true negatives. ^e^FP. false positives. ^f^ FN. false negatives.

Table S5. Data associated with the 49 false negative urine samples.

| Patient | Bacteria  identified^a^ | CFU/ml | Clinical info/comments | WBC^b^ | Bacterial counts (cels/µl)^b^ | AST^c^ |
| --- | --- | --- | --- | --- | --- | --- |
| 1 | mixed culture | - | - | 10.8 | 23.7 | No |
| 2 | mixed culture | - | - | 200.7 | 27.3 | No |
| 3 | yeast | 10^4^-10^5^ | Nitrite: no  Ab treatment | n.a. | 2.3 | No |
| 4 | yeast | 10^4^-10^5^ |  | n.a. | 10.7 | No |
| 5 | *Escherichia coli* | 10^4^-10^5^ | completed Ab treatment | 7.4 | 5.9 | Yes |
| 6 | *Klebsiella pneumoniae* | 10^4^-10^5^ | Nitrite: no  hematuria | 62.9 | 27.3 | Yes |
| 7 | *Enterococcus faecalis* | 10^4^-10^5^ | Nitrite: no | 174.4 | 27.3 | Yes |
| 8 | *Escherichia coli* | 10^3^-10^4^ | - | 1.6 | 20.2 | No |
| 9 | *Escherichia coli* | 10^3^-10^4^ | Nitrite: no | 2.5 | 21.4 | No |
| 10 | *Escherichia coli* | 10^3^-10^4^ | UTI symptoms | 2.6 | 10.7 | Yes  anamneses |
| 11 | *Escherichia coli* | 10^3^-10^4^ | - | 3.1 | 2.3 | Yes? |
| 12 | *Escherichia coli* | 10^3^-10^4^ | - | 3.1 | 17.8 | No |
| 13 | *Escherichia coli* | 10^3^-10^4^ | Nitrite: no | 4.4 | 7 | Yes? |
| 14 | *Escherichia coli* | 10^3^-10^4^ | 113 mikromol/L | 4.7 | 3.5 | Yes  anamneses |
| 15 | *Escherichia coli* | 10^3^-10^4^ | - | 5.2 | 15.4 | No |
| 16 | *Escherichia coli* | 10^3^-10^4^ | - | 6.2 | 3.4 | Yes? |
| 17 | *Escherichia coli* | 10^3^-10^4^ | - | 7.1 | 26.1 | No |
| 18 | *Escherichia coli* | 10^3^-10^4^ | - | 10.2 | 23.7 | No |
| 19 | *Escherichia coli* | 10^3^-10^4^ | - | 12 | 3.5 | Yes  recurrence |
| 20 | *Escherichia coli* ESBL | 10^3^-10^4^ | - | 12.7 | 7.1 | Yes  recurrence |
| 21 | *Proteus mirabilis* | 10^3^-10^4^ | - | 22.6 | 11.8 | Yes? |
| 22 | *Escherichia coli* | 10^3^-10^4^ | - | 54.2 | 9.5 | No |
| 23 | *Morganella morganii* | 10^3^-10^4^ | Leu 2+ | 258.1 | 2.3 | No |
| 24 | *Proteus mirabilis* | 10^3^-10^4^ | UTI symptoms  Nitrite: no | 355.2 | 4.7 | Yes  anamneses |
| 25 | *Escherichia coli* | 10^3^-10^4^ | 0 | n.a. | 2.3 | No |
| 26 | *Escherichia coli* | 10^3^-10^4^ | 0 | n.a. | 4.7 | No |
| 27 | *Escherichia coli* | 10^3^-10^4^ | 0 | n.a. | 4.7 | Yes? |
| 28 | *Pseudomonas species* | 10^3^-10^4^ | 0 | n.a. | 5.9 | No |
| 29 | *Escherichia coli* | 10^3^-10^4^ | 125 mikromol/L | n.a. | 7.1 | No |
| 30 | *Escherichia coli* | 10^3^-10^4^ | - | n.a. | 9.5 | No |
| 31 | *Klebsiella oxytoca* | 10^3^-10^4^ | - | n.a. | 9.5 | No |
| 32 | *Escherichia coli* | 10^3^-10^4^ | - | n.a. | 10.7 | No |
| 33 | *Escherichia coli* ESBL | 10^3^-10^4^ | - | n.a. | 15.4 | Yes  recurrence |
| 34 | *Citrobacter koseri* | 10^3^-10^4^ | - | n.a. | 15.4 | No |
| 35 | *Escherichia coli* ESBL | 10^3^-10^4^ | - | n.a. | 15.4 | Yes? |
| 36 | *Escherichia coli* | 10^3^-10^4^ | UTI symptoms Ab treatment | n.a. | 19 | Yes  anamneses |
| 37 | *Pseudomonas aeruginosa* | 10^3^-10^4^ | - | n.a. | 20.2 | Yes  requested |
| 38 | *Klebsiella oxytoca* | 10^3^-10^4^ | - | n.a. | 21.4 | No |
| 39 | *Escherichia coli* | 10^3^-10^4^ | - | n.a. | 22.6 | No |
| 40 | *Escherichia coli* | 10^3^-10^4^ | - | n.a. | 24.9 | No |
| 41 | *Escherichia coli* | 10^3^-10^4^ | Recurrent UTI/cystitis Nitrite: no | n.a. | 24.9 | Yes  anamneses |
| 42 | *Escherichia coli* | 10^3^-10^4^ | UTI symptoms Nitrite: no | n.a. | 24.9 | Yes  anamneses |
| 43 | *Escherichia coli* | 10^2^-10^3^ | UTI symptoms | 4.1 | 4.7 | Yes  anamneses |
| 44 | *Escherichia coli* | 10^2^-10^3^ | UTI symptoms | 7.1 | 7.1 | Yes  anamneses |
| 45 | *Escherichia coli* | 10^2^-10^3^ | - | 12 | 3.5 | Yes  recurrence |
| 46 | *Escherichia coli* | 10^2^-10^3^ | Nitrite: no | n.a. | 0 | No |
| 47 | *Escherichia coli* | 10^2^-10^3^ | - | n.a. | 1.1 | No |
| 48 | *Escherichia coli* | 10^2^-10^3^ | Kidney failure? | n.a. | 1.1 | Yes  anamneses |
| 49 | *Escherichia coli* | 10^2^-10^3^ | Nitrite: no | n.a. | 8.3 | Yes? |

28 males and 21 females. age range 31-98. flow cytometry cut-off of <30 cells/µl for the group where pregnant women and children are excluded (n=3261). ^a^Urine samples with 3 or more species were categorised as mixed culture. ^b^Bacterial counts and WBC=white blood cells estimated in flow cytometry analyses. ^c^AST= antimicrobial susceptibility testing was performed. yes; yes? for unclear reason or because of anamneses or recurrence.

Table S6. Predictive values of UFC compared to culture to detect relevant bacteriuria.

| **FCA cut-off** | **Group. n** | **SP^a^ %** | **PPV^b^ %** | **TP^c^** | **TN^d^** | **FP^e^** | **FN^f^** |
| --- | --- | --- | --- | --- | --- | --- | --- |
| 2000 | All patients, 4005 | 95.2 | 89.6 | 1014 | 2340 | 118 | 533 |
| 3500 |  | 97.3 | 93.4 | 939 | 2392 | 66 | 608 |
| 4000 |  | 97.8 | 94.4 | 915 | 2404 | 54 | 632 |
| 4500 |  | 98.3 | 95.4 | 900 | 2415 | 43 | 647 |
| 5000 |  | 98.5 | 96.1 | 880 | 2422 | 36 | 667 |
| 2000 | All women, 2657 | 93.2 | 87.3 | 729 | 1449 | 106 | 373 |
| 3500 |  | 96.3 | 92.1 | 675 | 1497 | 58 | 427 |
| 4000 |  | 96.9 | 93.2 | 657 | 1507 | 48 | 445 |
| 4500 |  | 97.6 | 94.4 | 645 | 1517 | 38 | 457 |
| 5000 |  | 98.0 | 95.3 | 633 | 1524 | 31 | 469 |
| 2000 | Pregnant women, 431 | 89.8 | 43.5 | 27 | 307 | 35 | 62 |
| 3500 |  | 94.7 | 56.1 | 23 | 324 | 18 | 66 |
| 4000 |  | 95.9 | 62.2 | 23 | 328 | 14 | 66 |
| 4500 |  | 97.4 | 71.0 | 22 | 333 | 9 | 67 |
| 5000 |  | 98.2 | 78.6 | 22 | 336 | 6 | 67 |
| 2000 | women, (excl. pregnant), 2226 | 94.1 | 90.8 | 702 | 1142 | 71 | 311 |
| 3500 |  | 96.7 | 94.2 | 652 | 1173 | 40 | 361 |
| 4000 |  | 97.2 | 94.9 | 634 | 1179 | 34 | 379 |
| 4500 |  | 97.6 | 95.6 | 623 | 1184 | 29 | 390 |
| 5000 |  | 97.9 | 96.1 | 611 | 1188 | 25 | 402 |
| 2000 | Men, 1348 | 98.7 | 96.0 | 285 | 891 | 12 | 160 |
| 3500 |  | 99.1 | 97.1 | 264 | 895 | 8 | 181 |
| 4000 |  | 99.3 | 97.7 | 258 | 897 | 6 | 187 |
| 4500 |  | 99.4 | 98.1 | 255 | 898 | 5 | 190 |
| 5000 |  | 99.4 | 98.0 | 247 | 898 | 5 | 198 |
| 2000 | Children (≤15 years), 313 | 98.7 | 91.7 | 33 | 225 | 3 | 52 |
| 3500 |  | 99.6 | 96.9 | 31 | 227 | 1 | 54 |
| 4000 |  | 99.6 | 96.9 | 31 | 227 | 1 | 54 |
| 4500 |  | 99.6 | 96.8 | 30 | 227 | 1 | 55 |
| 5000 |  | 99.6 | 96.6 | 28 | 227 | 1 | 57 |
| 2000 | Outpatients, 2430 | 95.5 | 90.4 | 628 | 1406 | 67 | 329 |
| 3500 |  | 97.6 | 94.2 | 587 | 1437 | 36 | 370 |
| 4000 |  | 98.0 | 95.0 | 569 | 1443 | 30 | 388 |
| 4500 |  | 98.3 | 95.7 | 558 | 1448 | 25 | 399 |
| 5000 |  | 98.6 | 96.5 | 549 | 1453 | 20 | 408 |
| 2000 | Hospitalised patients, 1575 | 94.8 | 88.3 | 386 | 934 | 51 | 204 |
| 3500 |  | 97.0 | 92.1 | 352 | 955 | 30 | 238 |
| 4000 |  | 97.6 | 93.5 | 346 | 961 | 24 | 244 |
| 4500 |  | 98.2 | 95.0 | 342 | 967 | 18 | 248 |
| 5000 |  | 98.4 | 95.4 | 331 | 969 | 16 | 259 |
| 2000 | All patients excl. pregnant women, 3574 | 96.1 | 92.2 | 987 | 2033 | 83 | 471 |
| 3500 |  | 97.7 | 95.0 | 916 | 2068 | 48 | 542 |
| 4000 |  | 98.1 | 95.7 | 892 | 2076 | 40 | 566 |
| 4500 |  | 98.4 | 96.3 | 878 | 2082 | 34 | 580 |
| 5000 |  | 98.6 | 96.6 | 858 | 2086 | 30 | 600 |
| 2000 | All patients excl. pregnant women + children, 3261 | 95.8 | 92.3 | 954 | 1808 | 80 | 419 |
| 3500 |  | 97.5 | 95.0 | 885 | 1841 | 47 | 488 |
| 4000 |  | 97.9 | 95.7 | 861 | 1849 | 39 | 512 |
| 4500 |  | 98.3 | 96.3 | 848 | 1855 | 33 | 525 |
| 5000 |  | 98.5 | 96.6 | 830 | 1859 | 29 | 543 |

^a^SP. specificity. ^b^PPV. positive predictive value. ^c^TP. true positives. ^d^TN. true negatives. ^e^FP. false positives. ^f^ FN. false negatives.
